# Exogenous L-Carnitine Promotes Plant Growth and Cell Division by Mitigating Genotoxic Damage of Salt Stress

**DOI:** 10.1038/s41598-019-53542-2

**Published:** 2019-11-21

**Authors:** Signem Oney-Birol

**Affiliations:** Department of Molecular Biology & Genetics, Faculty of Arts and Sciences, Burdur Mehmet Akif Ersoy University, Burdur, 15030 Turkey

**Keywords:** Cell-cycle exit, Abiotic

## Abstract

L-carnitine is a fundamental ammonium compound responsible for energy metabolism in all living organisms. It is an oxidative stress regulator, especially in bacteria and yeast and lipid metabolism in plants. Besides its metabolic functions, l-carnitine has detoxification and antioxidant roles in the cells. Due to the complex interrelationship of l-carnitine between lipid metabolism and salinity dependent oxidative stress, this study investigates the exogenous l-carnitine (1 mM) function on seed germination, cell division and chromosome behaviour in barley seeds (*Hordeum vulgare* L. cv. Bulbul-89) under different salt stress concentrations (0, 0.25, 0.30 and 0.35 M). The present work showed that l-carnitine pretreatment could not be successful to stimulate cell division on barley seeds under non-stressed conditions compared to stressed conditions. Depending on increasing salinity without pretreatment with l-carnitine, the mitotic index significantly decreased in barley seeds. Pretreatment of barley seeds with l-carnitine under salt stress conditions was found promising as a plant growth promoter and stimulator of mitosis. In addition, pretreatment of barley seeds with l-carnitine alleviated detrimental effects of salt stress on chromosome structure and it protected cells from the genotoxic effects of salt. This may be caused by the antioxidant and protective action of the l-carnitine. Consequently, this study demonstrated that the exogenous application of 1 mM l-carnitine mitigates the harmful effects of salt stress by increasing mitosis and decreasing DNA damage caused by oxidative stress on barley seedlings.

## Introduction

L-carnitine (4-N-trimethylammonium-3-hydroxybutyric acid, LC)^1^ is an endogenous ammonium compound; animals, bacteria, some yeast and fungi and plants naturally synthesise it from l-lysine and l-methionine amino acids^2–4^. The compound which is involved in energy metabolism, hormonal action, adaptation to stress and detoxifying functions^3,5–7^ has an important role in transporting long chain fatty acids from the cytosol into the mitochondrial membrane. Mitochondria are the cell’s powerhouse, breaking down sugar and synthesising of ATP to provide energy. In mammals, energy is created from activated fatty acids by mitochondrial β-oxidation of acyl-CoA NADH and FADH_2_ which enters the citric acid cycle (CAC) carried by carnitine shuttle from cytosol to mitochondria membrane^8,9^. The electron transport chain needs oxygen and NADP for the synthesis of ATP through oxidative phosphorylation. At the end of the CAC, oxygen is reduced to H_2_O leading to a reduction in the concentration of oxygen and in turn reducing the formation of reactive oxygen species (ROS)^5,10^. Oxidative stress is reduced by l-carnitine and its esters, which also regulates the activity of enzymes that defend the cell against oxidative damage^11^ and the levels of nitric oxide that influences cellular respiration^12^. In addition to protecting catalase and superoxide dismutase against 3-nitropropionic acid (3-NPA) induced neurotoxicity, l-carnitine also safeguards the activity of the mitochondrial enzyme, succinate dehydrogenase^13^.

Many types of plants, such as cereals and legumes contain l-carnitine, which can be found in various locations such as in leaves, as well as dry and germinating seeds^14^. The level of enzymatic activity of carnitine acyltransferase has been measured in plant tissues and chloroplasts^15–18^. Several studies measured the carnitine transferase activity from mitochondria mostly in pea chloroplasts and mung-bean hypocotyl^4^,^16^,^19–21^. Quantification studies of acylcarnitines and free carnitine in *Arabidopsis thaliana*, *Brassica napus*, *Linum usitatissinum* and *Nicotiana tobaccum*^6^ show a link between carnitine and lipid metabolism in plants. Lipids are essential to regulating the permeability of cell membranes and may provide plants with an effective means to resist salt^22–24^. Indeed, a number of studies describe changes to fatty acid, polar lipids and sterols as being influential in salt stress^4,6^. Although there is no direct evidence between carnitine and fatty acid metabolism, carnitine acyltransferase enzymes is formed from carnitine esters from acyl CoAs which is responsible from the mitochondrial β-oxidation in the avocado plant and pea seedlings^14,25–28^.

Salinity presents a serious environmental problem as it affects life cycle parameters such as plant fertility, growth and production. Salinity affects approximately 20% of the world’s total agricultural land^29–32^. Salinity causes oxidative stress, which in turn modifies the composition of fatty acids and the lipid content of organisms. The ability of plants to adapt to different environmental conditions is directly affected by the composition of fatty acids and cell membrane lipids^33–36^. Depending on plant species and intensity of the salt stress to which the plant is exposed, lipids are key to regulating selective cell membrane permeability^37,38^. According to Shaya-Khmetova *et al*.^39^, after applying exogenous salt stress to wheat species, the total lipid content had decreased in a week. The change in the concentration of salt modulates the activity of ion-transporting proteins^40^ and the synthesis of osmolytes^41^, reflecting changes in the fatty acid composition of cell membrane lipids and lipid metabolism^42^. Furthermore, the levels of carnitine correlates with tolerance to abiotic stresses in plants^30^. Considering the positive effects of l-carnitine on lipid metabolism, fatty acid composition, cell membrane permeability and apoptosis under biotic and abiotic stress this study designed to determine how mitotic cycle is affected by l-carnitine and how l-carnitine alleviate detrimental effects of different concentrations of exogenous salt stress. The study was designed to determine whether l-carnitine could be effective to increase cell division and might be suppressor on programmed cell death under salt stress conditions; it also evaluates whether the compound can alleviate genotoxic effects of salt stress on DNA level and the chromosome structure in the meristematic root tip cells of barley (*Hordeum vulgare* L.).

## Results

### Effects of L-carnitine on cell division under salt stress

**Figure 1:**
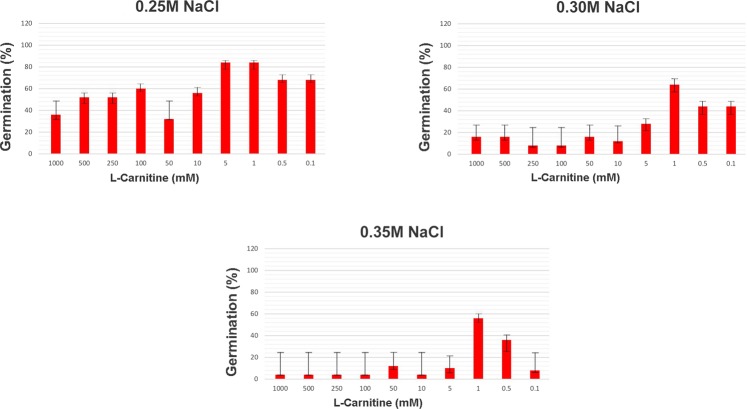
Germination percentage of barley seeds with growth of cotyledons with respect to the total number of emerged seedlings after 7 days. Data were presented as the mean values obtained from three independent experiments covered with 50 seeds. The error bars indicate the standard deviation (±SD).

A preliminary study was performed to determine appropriate concentrations of NaCl to use; 0.25 M, 0.30 M and 0.35 M were deemed suitable to initiate salt stress responses in barley seeds. The salt concentrations preventing germination of seeds to a great extent (above 50% germination rate) were used to determine the NaCl concentrations. As a result of the preliminary study to identify which dose of the l-carnitine is effective for alleviating salt stress from 1000 mM to 0.1 mM concentrations were tested. As shown in the Fig. 1, the percentage of seed germination showed the same effect for 5 mM (84%) and 1 mM (84%) in 0.25 M salt. Depending on the increased salt stress concentration, the percentage of the germination regularly decreased in the 5 mM LC level (28% at 0.30 M and 10% at 0.35 M). Also, the experimental studies showed that l-carnitine solution must be prepared before used to get the same effects on seed germination. Finally, 1 mM l-carnitine concentration was found to be the most effective dose to offset the damage of salt stress stimulated to seed germination and the regulation of plant growth.

L-carnitine pretreatment (positive control, 1 mM LC) could not be successful to stimulate cell division on barley seeds by alone. Generally, mitotic index of barley seeds germinated in distilled water (negative control, 0 M NaCl) showed a higher rate (0.17) than pre-treated seeds group with l-carnitine (0.07) (Table 1). In addition, the number of anaphase and telophase cells were very low in both groups. However, most of the cells were observed in prophase stage in the negative control group. There is a statistically significant difference was determined in the prophase and metaphase indices between pre-treated and untreated groups with LC (*p* ≤ 0.05) (Table 1). Depending on the increasing salt stress concentration, the mitotic index significantly decreased in barley seeds. For instance, although the mitotic index value was calculated 0.17 in negative control group, it was 0.16 at 0.25 M NaCl, 0.14 at 0.30 M NaCl and 0.12 at 0.35 M NaCl concentrations. However, mitotic index of the pretreatment with l-carnitine under salt stress conditions was found promising as a plant growth promoter. In other words, pretreatment seeds with 1 mM l-carnitine to overcome detrimental effects of salt stress on cell division had effective results with the 0.28 mitotic index value at 0.25 M NaCl, 0.24 at 0.30 M NaCl and 0.14 at 0.35 M salinity conditions (Table 1). As seen from the phase indices after pretreatment with l-carnitine, the mitotic cycle was stimulated by increasing the prophase stage under salt stress conditions.

**Table 1:** Mitotic index and phase indices of root tip cells of *H. vulgare* L. after pretreatment with 1 mM l-carnitine and unpretreated group germinated in distilled water (negative control; 0 M NaCl) were shown under various salt stress concentrations.

Treatment	Mitotic Index (MI)	Mitotic Index (%)	Prophase Indice (I_p_)	Metaphase Indice (I_M_)	Anaphase Indice (I_A_)	Telophase Indice (I_T_)
0 M NaCl (Negative Control)	*0.17 ± 0.04^b^	17	0.12 ± 0.04^bc^	0.04 ± 0.02^d^	0.00 ± 0.00^a^	0.00 ± 0.00^a^
0.25 M NaCl	0.16 ± 0.01^ab^	16	0.09 ± 0.04^b^	0.02 ± 0.01^abc^	0.00 ± 0.00^a^	0.00 ± 0.00^a^
0.30 M NaCl	0.14 ± 0.01^ab^	14	0.05 ± 0.01^a^	0.01 ± 0.00^a^	0.00 ± 0.00^a^	0.00 ± 0.00^a^
0.35 M NaCl	0.12 ± 0.01^a^	12	0.03 ± 0.01^a^	0.01 ± 0.01^ab^	0.00 ± 0.00^a^	0.00 ± 0.00^a^
0 M NaCl + 1 mM LC (Positive Control)	0.07 ± 0.01^a^	7	0.04 ± 0.01^a^	0.02 ± 0.01^c^	0.00 ± 0.00^a^	0.00 ± 0.00^a^
0.25 M NaCl + 1 mM LC	0.28 ± 0.08^c^	28	0.19 ± 0.05^d^	0.02 ± 0.01^bc^	0.01 ± 0.01^a^	0.01 ± 0.01^a^
0.30 M NaCl + 1 mM LC	0.24 ± 0.01^c^	24	0.16 ± 0.02 ^cd^	0.02 ± 0.01^c^	0.02 ± 0.01^b^	0.02 ± 0.01^c^
0.35 M NaCl + 1 mM LC	0.14 ± 0.02^ab^	14	0.05 ± 0.01^a^	0.01 ± 0.00^abc^	0.01 ± 0.01^a^	0.01 ± 0.01^b^

*Values with insignificant difference (p ≤ 0.05) for each column are indicated with same letters (means ± SD).

### Effects of L-carnitine on chromosome structure under salt stress

**Figure 2:**
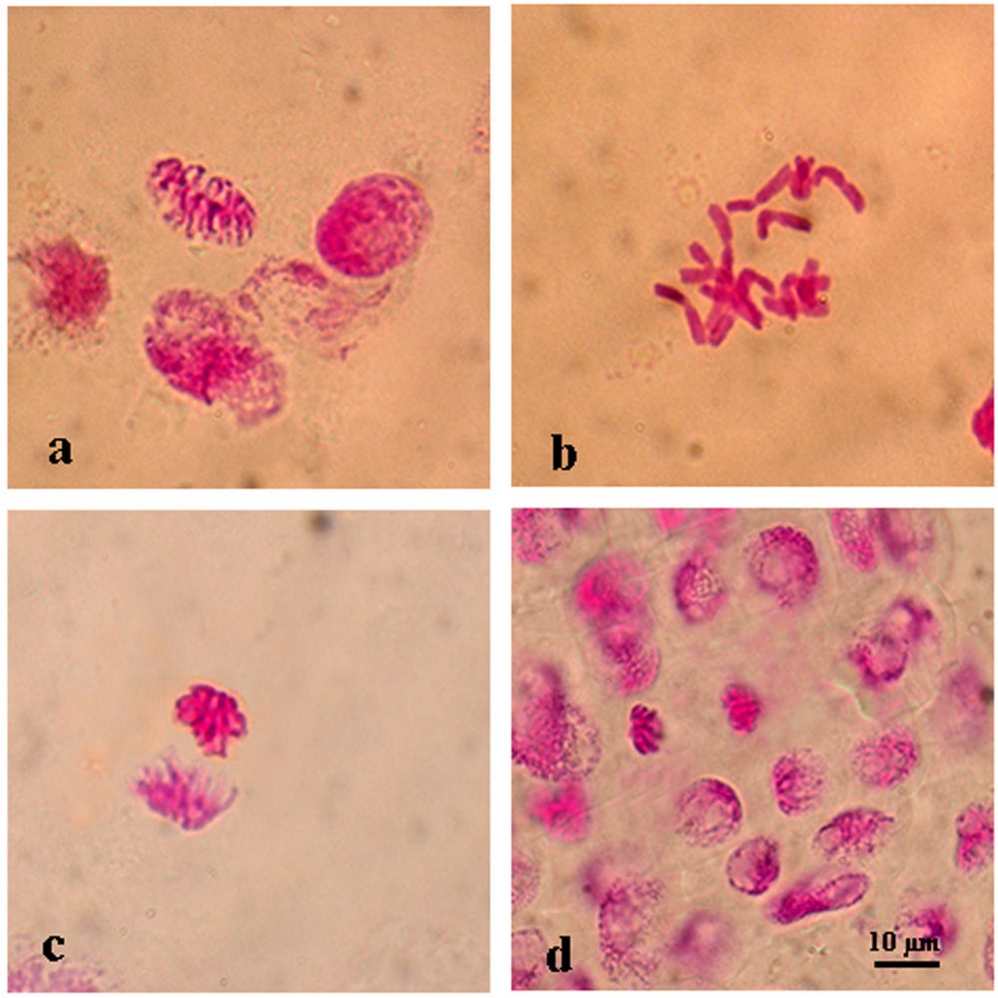
Normal mitotic chromosome structure in the meristematic root tip cells of barley (*H. vulgare* L. cv. Bulbul-89) seeds without pretreatment with l-carnitine under different salt stress concentrations. Prophase (**a**), metaphase 2n = 24 (**b**), anaphase (**c**), telophase (**d**). Scale bar: 10 µm.

**Figure 3:**
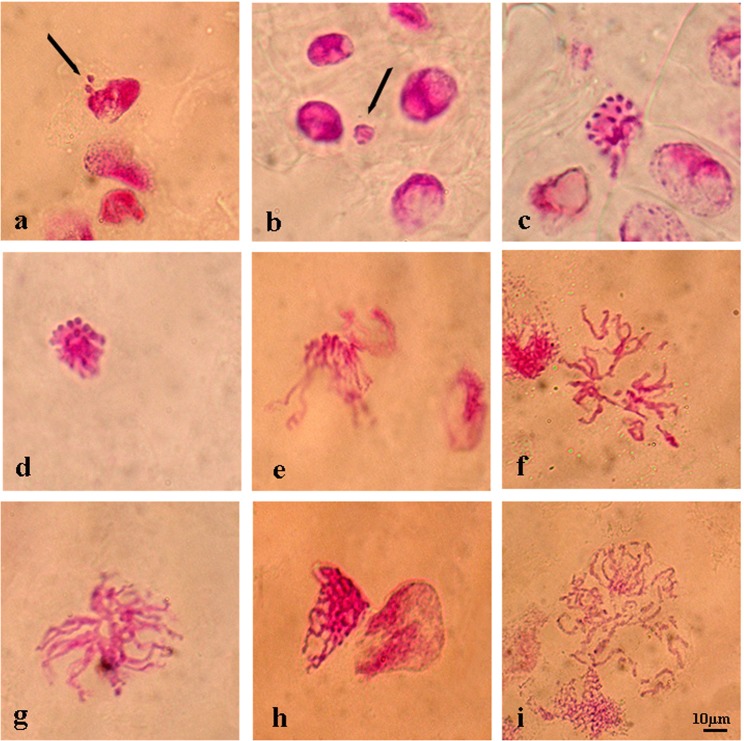
Representative photos of prophase level in the meristematic root tip cells of barley (*H. vulgare* L. cv. Bulbul-89) seeds pretreatment with and without l-carnitine under different salt stress concentrations. Micronucleus (**a**,**b**), granulation (**c**,**d**), disorderly prophase, (**e–i**). Scale bar: 10 µm.

**Figure 4:**
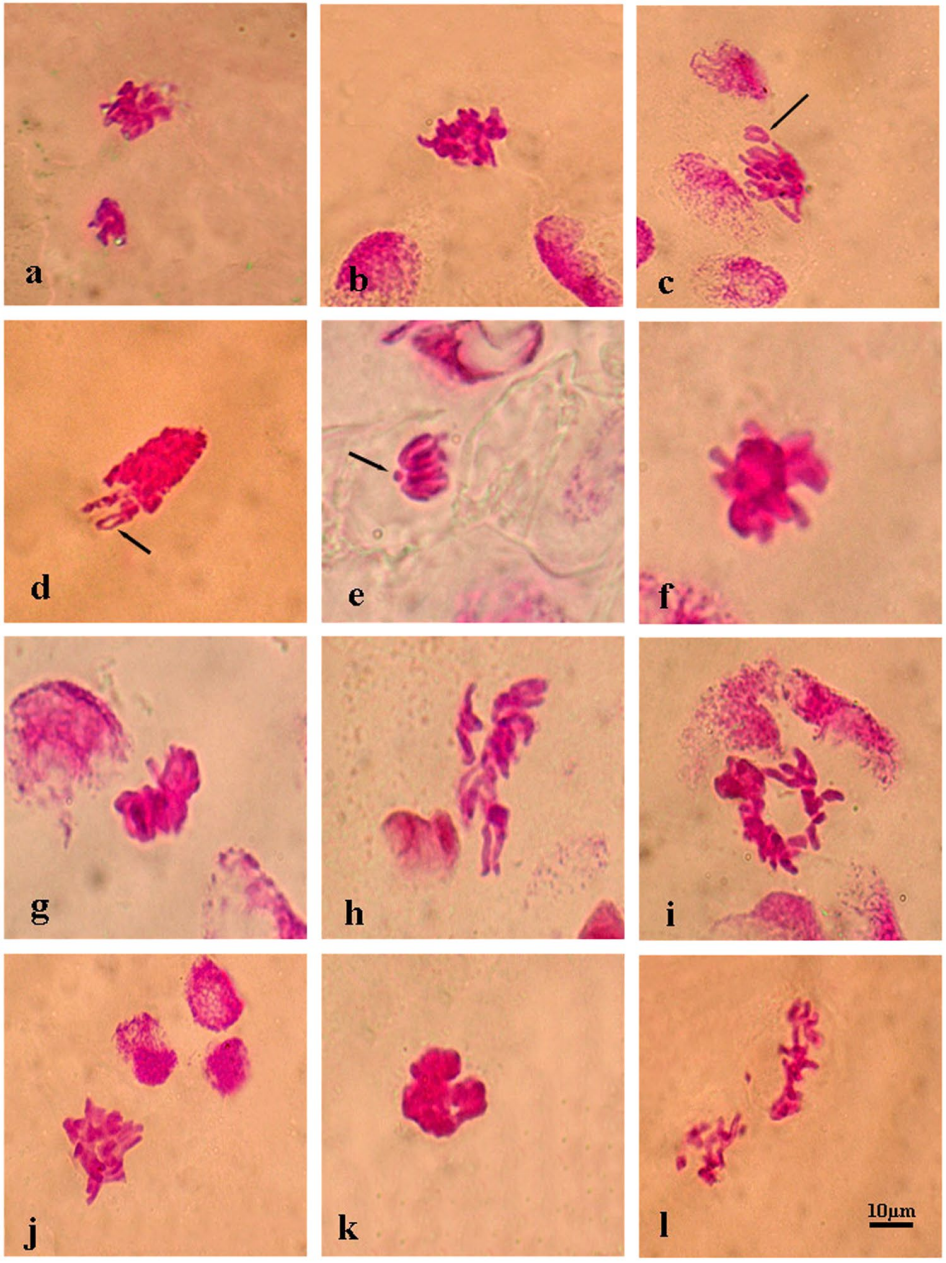
Representative photos of metaphase level in the meristematic root tip cells of barley (*H. vulgare* L. cv. Bulbul-89) seeds pretreatment with and without l-carnitine under different salt stress concentrations. Disrupted equatorial plate (**a**,**h**,**l**), ring chromosomes (**c–e**), stickiness (**b,f,g,i–k**) (→ represent aberrant chromosome). Scale bar: 10 µm.

**Figure 5:**
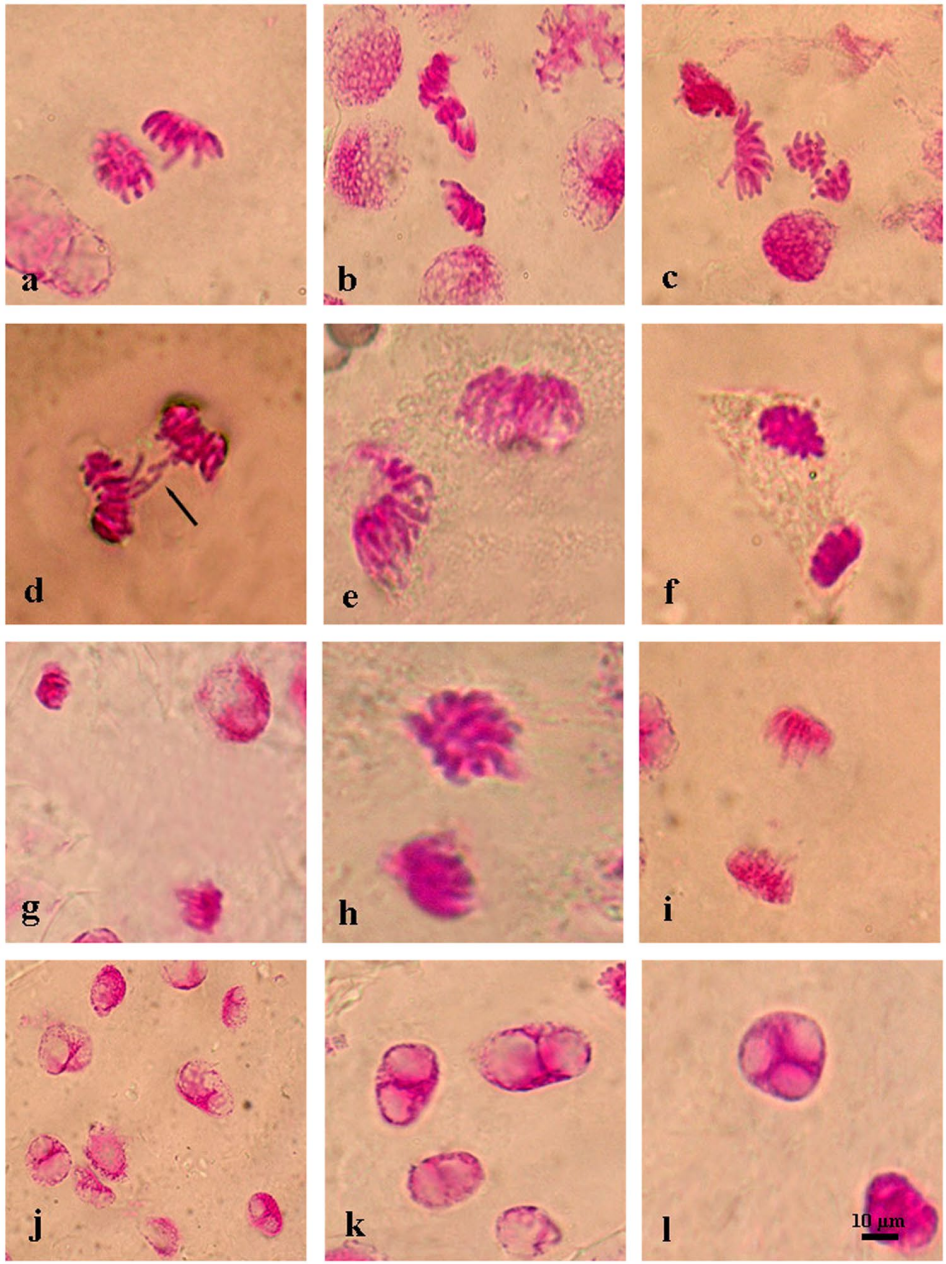
Representative photos of anaphase level in the meristematic root tip cells of barley (*H. vulgare* L. cv. Bulbul-89) seeds pretreatment with and without l-carnitine under different salt stress concentrations. Vagrant chromosomes (**a,c,d**), spindle abnormalities in anaphase (**b,c**), anaphase bridge (**d**), fault polarization (**e–h**), alignment anaphase (**i**), binucleated cells (**j,k**), multinucleated cell (**l**) (→ represent aberrant chromosome). Scale bar: 10 µm.

The negative control (distilled water, 0 M NaCl) group showed normal mitotic cells (Table 2, Fig. 2) and regular formation of mitotic chromosome number (2n = 2x = 14) while different types of chromosomal aberrations were observed in the root tips of positive control (1 mM LC + 0 M NaCl) group cells with the rate of 0.08. However, barley seeds exposed to salt stress (0.25, 0.30, 0.35 M NaCl) exhibited with different types of chromosome aberration indices in different phases of mitosis (Table 2, Figs 3–5). The genotoxicity index (*I*_*G*_) depending on increasing concentration of salt stress was significantly (*p* ≤ *0.05*) higher than both control groups. The frequency of chromosomal aberrations was 0.23 at 0.25 M, 0.51 at 0.30 M and 0.72 at 0.35 M NaCl concentrations. Pretreatment barley seeds with 1 mM l-carnitine showed regenerative impact on chromosome aberrations under salt stress. Genotoxicity index was completely decreased depending on increasing salt stress concentration (Table 2). These aberrations were found 0.06 (6%) at 0.25 M, (20%) at 0.30 M and 0.15 (15%) at 0.35 M. In all tested salt concentrations, pretreatment with l-carnitine showed effective results to alleviate mutational effects on the chromosome structure, and l-carnitine pretreatment was significantly successful to inhibit detrimental effects of salt stress on chromosome behaviour.

**Table 2:** Genotoxicity index (*IG*) and chromosome aberrations of the root tip cells of *H. vulgare* L. after pretreatment with 1 mM l-carnitine and unpretreated group germinated in distilled water (negative control; 0 M NaCl) were shown under various salt stress concentrations.

Treatment	Genotoxicity Index (I_G_)	Disorderly Prophase	Stickiness	Ring Chromosome	Micronucleus	Alignment Anaphase	Fault polarization	Anaphase /Telophase Bridge	Vagrant Chromosome	Multinucleated cells
0 M NaCl (Negative Control)	*0.00 ± 0.00^a^	0.00 ± 0.00^a^	0.00 ± 0.00^a^	0.00 ± 0.00^a^	0.00 ± 0.00^a^	0.00 ± 0.00^a^	0.00 ± 0.00^a^	0.00 ± 0.00^a^	0.00 ± 0.00^a^	0.00 ± 0.00^a^
0.25 M NaCl	0.23 ± 0.08^d^	0.07 ± 0.01^a^	0.00 ± 0.00^a^	0.01 ± 0.01^b^	0.00 ± 0.00^ab^	0.00 ± 0.01^ab^	0.00 ± 0.01^ab^	0.00 ± 0.01^a^	0.00 ± 0.00^a^	0.00 ± 0.00^a^
0.30 M NaCl	0.51 ± 0.03^e^	0.12 ± 0.01^b^	0.00 ± 0.00^a^	0.02 ± 0.01^c^	0.00 ± 0.00^a^	0.00 ± 0.00^a^	0.00 ± 0.01^ab^	0.00 ± 0.00^a^	0.00 ± 0.00^a^	0.00 ± 0.00^a^
0.35 M NaCl	0.72 ± 0.07^e^	0.09 ± 0.02^b^	0.00 ± 0.00^a^	0.01 ± 0.01^c^	0.00 ± 0.00^a^	0.00 ± 0.00^a^	0.00 ± 0.00^b^	0.00 ± 0.01^a^	0.00 ± 0.00^a^	0.00 ± 0.00^a^
0 M NaCl + 1 mM LC (Positive Control)	0.08 ± 0.01^ab^	0.01 ± 0.01^a^	0.01 ± 0.00^a^	0.00 ± 0.00^a^	0.00 ± 0.00^a^	0.00 ± 0.00^a^	0.00 ± 0.00^a^	0.00 ± 0.00^a^	0.00 ± 0.00^a^	0.06 ± 0.00^bc^
0.25 M NaCl +1 mM LC	0.06 ± 0.04^a^	0.01 ± 0.02^a^	0.04 ± 0.01^b^	0.00 ± 0.00^a^	0.01 ± 0.01^b^	0.00 ± 0.00^a^	0.01 ± 0.01^ab^	0.01 ± 0.01^a^	0.01 ± 0.01^b^	0.04 ± 0.02^b^
0.30 M NaCl +1 mM LC	0.20 ± 0.04^cd^	0.01 ± 0.01^a^	0.03 ± 0.01^b^	0.01 ± 0.01^b^	0.01 ± 0.00^b^	0.02 ± 0.00^b^	0.01 ± 0.01^b^	0.00 ± 0.00^a^	0.00 ± 0.00^a^	0.16 ± 0.04^d^
0.35 M NaCl +1 mM LC	0.15 ± 0.05^bc^	0.02 ± 0.01^a^	0.04 ± 0.00^b^	0.01 ± 0.00^b^	0.01 ± 0.00^b^	0.02 ± 0.01^a^	0.01 ± 0.01^b^	0.01 ± 0.02^a^	0.01 ± 0.01^b^	0.09 ± 0.02^c^

*Values with insignificant difference (P ≤ 0.05) for each column are indicated with same letters (means ± SD).

**Figure 6:**
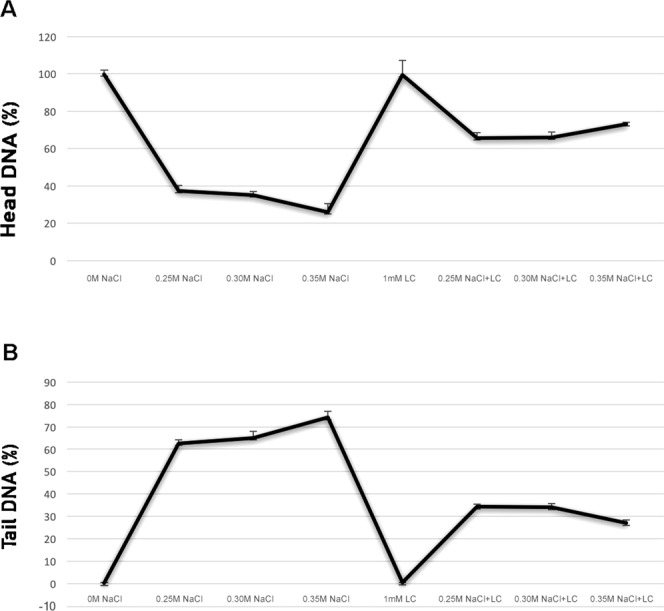
Comparison of DNA damage using Comet assay in the meristematic root tip cells of barley (*H. vulgare* L. cv. Bulbul-89) seeds pretreatment with and without l-carnitine under different salt stress concentrations. (**A**) Percentage of head DNA, (**B**) Percentage of tail DNA. Error bars indicated standard deviation (±SD), n = 200, (*p ≤ 0.05).

**Figure 7:**
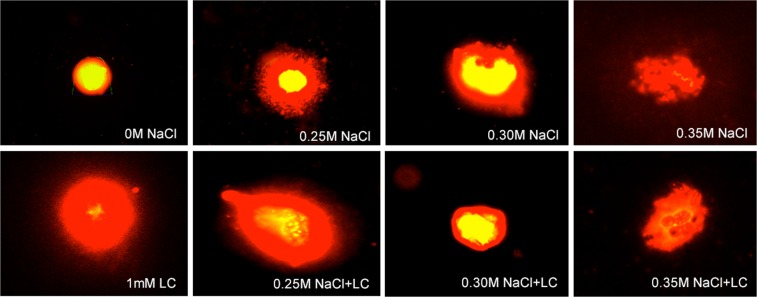
Representative photos of DNA comets in the meristematic root tip cells of barley (*H. vulgare* L. cv. Bulbul-89) seeds observed in comet assay pretreatment with and without l-carnitine under different salt stress concentrations.

The most common monitored aberrations were also disorderly prophase (Fig. 3), stickiness (Fig. 4) and multinucleated cells (Fig. 5j–l) in seeds pre-treated with l-carnitine and different salt stress concentrations. All multinucleated cells were encountered in the positive control and pre-treated seeds with l-carnitine while seeds germinated in salt stress concentrations and distilled water did not showed any multinucleated cells. In addition, micronucleus (Fig. 3a,b), granulation (Fig. 3c,d), disrupted equatorial plate (Fig. 4a,h), ring chromosomes (Fig. 4c,d), vagrant chromosomes (Fig. 5a,c,d) and spindle abnormalities in anaphase (Fig. 5b,c), anaphase and telophase bridges (Fig. 5d), fault polarization (Fig. 5e–h), alignment anaphase (Fig. 5i) were also observed and noticed as statistically significant (*p* ≤ *0.05*) compared to most common chromosome aberrations. Also, screening the DNA damage on nuclei to find more proof about genotoxicity, all threated samples were examined using Comet Assay (Table 3). Based on the genotoxicity index, depending on the salt stress concentration from 0 to 0.35 M, the percentage of tail DNA was increased from 0.17 ± 0.02 to 74.24 ± 2.70 (Fig. 6). Indeed, the 0 M NaCl concentration showed no head DNA (99.83%) damage. However, 0.35 M NaCl concentration showed significant differences on head DNA level (25.76%) (*p* ≤ *0.05*). On the other hand, the application of 1 mM l-carnitine to barley seeds showed almost no damage on tail DNA (0.45%). Furthermore, the most protective role of l-carnitine on DNA level on barley seeds was observed in the highest concentration (0.35 M) of salt, after pretreatment with l-carnitine the percentage of tail DNA decreased almost three-fold from 74.24 ± 2.70 to almost 27.03 ± 1.30. Figure 7 also shows the changes on the nucleus on DNA damage due to the concentration levels.

**Table 3:** Comet assay (head DNA %, tail DNA %) scores of the root tip cells of *H. vulgare* L. after pretreatment with 1 mM l-carnitine and unpretreated group germinated in distilled water (negative control; 0 M NaCl) were shown under various salt stress concentrations.

Treatment	Head DNA (%)	Tail DNA (%)
0 M NaCl (Negative Control)	99.83 ± 2.22^a^	0.17 ± 0.02^a^
0.25 M NaCl	37.34 ± 2.77^abc^	62.70 ± 1.37^b^
0.30 M NaCl	34.89 ± 2.08^ab^	65.11 ± 2.77^b^
0.35 M NaCl	25.76 ± 4.72^ab^	74.24 ± 2.70^c^
0 M NaCl + 1 mM LC (Positive Control)	99.55 ± 7.84^a^	0.45 ± 0.08^ab^
0.25 M NaCl + 1 mM LC	65.66 ± 2.82^bcd^	34.34 ± 1.09^ab^
0.30 M NaCl + 1 mM LC	65.92 ± 2.81^bcd^	34.08 ± 1.56^ab^
0.35 M NaCl + 1 mM LC	72.97 ± 1.03^cd^	27.03 ± 1.30^ab^

*Values with insignificant difference (*p* ≤0.05) for each column are indicated with same letters (means ± SD).

## Discussion

In the present study, cytogenetic and genotoxic effects of l-carnitine as a plant growth promoter germinated in different salt stress concentrations (0, 0.25, 0.30 and 0.35 M) was reported by determining the putative effects on seed germination, cell division and DNA damage in the meristematic root tip cells of *H. vulgare* L.

Pretreatment of barley seeds with l-carnitine made a mitodeppresive effect on cell cycle compared to the negative control group germinated seeds in distilled water. While mitotic index was counted 17% in the negative control group cells it was observed in the positive control group cells as 7% (see Tables 1 and 2). Chromosomal alterations were not observed in the root tip cells of barley seeds germinated in distilled water and the genotoxicity index was 0.00% (see Table 2, Fig. 2). However, the barley seeds pre-treated with 1 mM l-carnitine showed 8% genotoxicity index and different types of chromosome aberrations such as multinucleated cells and disorderly prophase (see Table 2, Fig. 3). According to the comet assay genotoxicity results, percentage of tail DNA in positive control group cells increased 2.65-fold compared with the negative control group cells. Furthermore, 1 mM concentration of l-carnitine showed more genotoxic effects under unstressed conditions which might be cytotoxic on the mitotic chromosome structure in barley seeds. Otherwise, there are limited reports about l-carnitine levels in plant metabolism^43,44^. The only report for Panter and Mudd^45^ presented endogenous l-carnitine levels in some higher plants and it was observed that barley seeds did not contain any level of l-carnitine. Hence, barley seeds were used to determine the exogenous effects in all studied parameters pre-treated with l-carnitine for 24 hours. These results indicated that exogenous l-carnitine was not effective to stimulate cell cycle for mitosis in the absence of stress (see Table 1).

Though there are numerous studies of exogenous l-carnitine uptake under stress conditions in yeast^46,47^, bacteria^48–50^ and animals^51^, in plants most of the studies were performed about the endogenous level of l-carnitine content^4,29,52,53^, whereas this study reported for the first-time alleviative effects of exogenous l-carnitine on cell division and DNA damage under salt stress conditions.

Cytogenetic monitoring and Comet Assay are the most effective method to determine species-specific effects on DNA level. These allows the researchers to understand the cytogenetic changes via mutagenesis. The mitotic index also is a cytogenetic monitoring test which measures the proliferation of mitotic cells (M phase) in the cell cycle and its inhibition could be scored as cellular death^54,55^. There are numerous studies showing that l-carnitine decreased cell apoptosis, and death and chromosomal alterations in mammals^56,57^ and mice^58^. Furthermore, cytoprotective effects of l-carnitine was frequently investigated in human metabolism under oxidative stress conditions^57,59^.

Salinity is a global and major problem reducing agricultural crop production all over the world. It is reported that ~50% of the world’s arable land will be affected from the increasing soil salinity by 2050^60,61^. Salinity also has a very strong relationship with osmotic and oxidative stress acting osmolytes, reactive oxygen species and membrane proteins^53,62–64^. Salt accumulation in the root systems generates osmotic stress by inhibiting water and essential elements uptake^65^ and high concentration of salt stress cause repressive effects on DNA, RNA and protein synthesis, cell cycle progression, seed germination, plant growth and production^66,67^. To survive under salt stress, plants adapt their mechanism via activating genes and protein kinases associated with stresses, signal transduction involved in cell division^24,68^. To increase plant resistance to salt stress, researchers or breeders focus on identifying new nutrients and fertilizers given to plants exogenously for improving plant production. As an exogenous supplement in plant metabolism, these findings suggest that l-carnitine could be a potential salt stress inhibitor stimulating progression of cell cycle via increasing mitosis under salt stress conditions (see Table 1), because l-carnitine has antioxidant action by activating antioxidant enzymes responsible from the synthesis of protective molecules reported by numerous studies in mammals under stress conditions^59^. Similarly, antioxidants influence specific cell cycle transitions to control cell cycle and protect plant cells from oxidative stress damages^53,69–71^. According to Charrier *et al*.^29^ exogenous carnitine stimulates development and protects *Arabidopsis thaliana* seedlings from the oxidative and salt stress damage^29^. Likewise, the results of this study, as a new plant growth stimulator by promoting seed germination, increasing cell division and decreasing the damage on DNA caused by salt stress, 1 mM l-carnitine is recommended as the appropriate dose to protect cells from the oxidative damage.

On the other hand, increasing salt stress concentrations breaks down the chromosomal structure in the root tip cells of barley seeds. Numerous cytogenetic studies reported that mutagenic effects of high levels salt stress on plant cell metabolism result from possible structural or numerical abnormalities on chromosomes^72–74^. Similarly, the highest chromosome alteration percentage was observed in the highest salt stress concentration (84%, 0.35 M) in this study. The sensitivity of the genotype to salt stress was statistically significant and different. Disorderly prophase (12 and 9%, respectively in 0.30 and 0.35 M NaCl) was the most common chromosome aberration in barley seeds germinated in salt stresses. Moreover, ring chromosomes (2%) (see Table 2, Fig. 4c,d) were the second significant aberration in 0.30 M salt stress concentration. Salinity side effects on chromosome structure were dependent to salt stress level, where a low level of salt stress has fewer side effects in contrast to high level stress application in all studied treatment group cells (see Table 2). Pretreatment with l-carnitine also significantly decreased the percentage of the genotoxicity index or chromosome aberrations and DNA damage on tail DNA in the meristematic root tips cells of barley (by ~5-fold in 0.25 M, ~4-fold in 0.30 M and ~6-fold in 0.35 M NaCl) compared to the absence of l-carnitine under salt stress (see Table 3). This could also be due to the regulation of the protective effect as well as DNA repair capability of l-carnitine^58,75,76^. These findings showed that l-carnitine was effective at different degrees to alleviate detrimental effects of salt stress on chromosome structure (see Table 2). However, different types of chromosomal alterations were also counted in pretreatment with 1 mM l-carnitine such as micronucleus, stickiness, alignment anaphase, fault polarization, anaphase/telophase bridges, vagrant chromosomes and multinucleated cells in interphase cells in the meristematic root tip cells of barley seeds under salt stress conditions. Interestingly, multinucleated cells were only observed in the pre-treated l-carnitine groups both the absence of salt stress and under salt stress concentrations (see Table 2). So, it may result from the l-carnitine and may cause formation of multinucleated cells in interphase level. Similarly, some researchers reported that a significant decrease in DNA damage and chromosome aberrations pre-treated with l-carnitine alleviates negative effects of oxidative stress^56,77^ and inhibition of tumor growth^78,79^ and cancer cell death^80^ in animal cells. According to Missihoun *et al*.^81^ the regulation of carnitine biosynthesis was affected by the salt stress levels in *A. thaliana* wild and transgenic plants that expression level of the betaine aldehyde dehydrogenase genes has an important role on the early growing stage of the seedlings to mitigate the salt stress in young tissues. Compared to the results of this study, it was determined that l-carnitine might have a cytoprotective effect to balance the overexpression level of the genes depending on increasing levels of salt stress. Due to the physiological functions of the carnitine and other betaines such as protection of enzyme structure and increasing membrane stability depending on the stress level have been reported as non-toxic even in high concentrations^82^. Otherwise, positive control group (1 mM l-carnitine) found to be genotoxic on the barley chromosome structure compared to the negative control group (0 mM l-carnitine). However, the presented study here suggests that l-carnitine has a protective effect on chromosome structure and DNA level against the genotoxic effects under the salt stress especially in high concentrations in barley cells.

In summary, the results of this study imply that the level of l-carnitine pretreatment (1 mM) was species specific under the tolerance levels to salt stress of *H. vulgare* L. Testing the putative cytoprotective effects of l-carnitine as an exogenous supplement were identified on cell cycle proliferation, genotoxicity and the level of DNA damage under salinity. A deeper understanding of these protective role of l-carnitine on DNA level related to putative salt stress sensors might help marker assisted breeding and genetic engineering to cope with various abiotic stresses.

## Methods

### Plant materials, experimental design and cytogenetical analysis

Barley (*Hordeum vulgare* L., 2n = 14, Fig. 2b) is one of the world’s oldest and most often grown cultivated crop plant. Its success and popularity for agriculture is based on its high adaptive capacity to withstand abiotic stress. Because of its innate ability to endure drought, fungal infection and salinity, barley is a model plant; thus, it is an ideal model organism for in biological research to study plant stress responses. This study used barley (*H. vulgare* L. cv. Bulbul-89) seeds to explore the cytogenetic response of l-carnitine (C_7_H_15_NO_3_, MA: 161.2 g/mol) in salt stress conditions.

Three biological replicates of each twenty-five uniformly sized barley seeds were prepared for each application by pre-treating with 1 mM of l-carnitine or distilled water (negative control), then leaving them at room temperature for 24 h. Then the solutions were removed and the seeds were vacuum-dried^83^. For each application, the seeds were put into 10 cm petri dishes and covered with two sheets of filter paper; sheets were moistened with 10 ml of distilled water (0 M salt) and the others with one of the predetermined concentrations of salt (0.25 M, 0.30 M or 0.35 M). To allow the seeds to germinate, the petri dishes were incubated at 20 ± 1 °C for 7 days. The root tips of the germinated seeds were cut off once they were 1–1.5 cm long, and they were pre-treated for 4 hours with paradichlorobenzene. The root tips were fixed in Carnoy solution [(ethanol (99%): glacial acetic acid (3:1)] for 24 h. Then they were stored at 4 °C in 70% ethanol until required. The root tips were hydrolyzed for 17 min. using 1 N HCl and stained using the Feulgen protocol for at least 1 h and before squashing the tips on slides with a little 45% acetic acid. At least 3.000 cells per treatment (1.000 per slide) were evaluated, and the mitotic index, phase indices (I) of dividing cells and chromosome aberrations were scored. To calculate the mitotic index (%), the number of cells undergoing mitotic division was divided by the total number of cells × 100^84^. For a more detailed evaluation of cell division, we calculated the indices (I) of the separate phases, prophase (*IP*), metaphase (*IM*), anaphase (*IA*) and telophase (*IT*). In accordance with Ivanova *et al*.^85^ the following formula was used for the phase indices:

$${{\rm{I}}}_{phase}( \% )={\rm{the}}\,{\rm{cell}}\,{\rm{number}}\,{\rm{of}}\,{\rm{respective}}\,{\rm{phase}}/{\rm{the}}\,{\rm{total}}\,{\rm{number}}\,{\rm{of}}\,{\rm{divided}}\,{\rm{cells}}\times 100$$

The effects of l-carnitine and the cytotoxicity of salt stress were determined by scoring the number of aberrant cells by the total number of divided cells. The percentage of aberrations was calculated as follows:

$${\rm{Genotoxicity}}\,{\rm{index}}( \% )={\rm{Number}}\,{\rm{of}}\,{\rm{any}}\,{\rm{kind}}\,{\rm{of}}\,{\rm{abnormality}}\,{\rm{observed}}/{\rm{total}}\,{\rm{number}}\,{\rm{of}}\,{\rm{cells}}\,{\rm{observed}}\times {\rm{100}}$$

An Olympus CX- 41 research microscope (100X objective) was used to observe the chromosome structure; they were photographed using a C–5060 WZ camera.

### Commet assay

Microscope slides were pre-heated and coated with 1% normal melting point agarose and dehydrated at room temperature. The meristematic root tips of barley seedlings were chopped in 600 μl nucleus isolation buffer (4 mM MgCl_2_.6H_2_O, 0.5% Triton^TM^X-100 and 0.2 M Tris, pH 7.5) for 30 s. The buffer containing nuclei was transferred to a clean microcentrifuge tubes for each treatment and the tubes were centrifuged at 12,000 rpm for 15 min. at 4 °C. Following centrifugation, 50 μl of suspension of nuclei were placed on each slide mixed with 50 μl 1% low melting point agarose at 55 °C, covered with 20 × 50 mm cover glass and stated on ice for 5 min. Then, the samples were subjected to electrophoresis buffer in high salt solution (300 mM NaOH, 1 mM EDTA, pH > 13) for 30 min. at 4 °C. After that, they were exposed to 25 V for 20 min. and 300 mA and cooled on ice for 5 min. For neutrolization step, the slides were kept in 100 mM Tris-HCl for 5 min. Each slide stained with 70 μl ethidium bromide solution and kept at 4 °C for 5 min. to photograph the DNA damage on nuclei under fluorescence microscope on 200 cells/comets for each concentration using OpenComet v1.3.1 Software.

### Statistical analysis

To compare the samples from the untreated group (control) with those pre-treated with l-carnitine (1 mM), ANOVA analysis was performed using MiniTab 17^86^ and SPSS 22^87^ software. The non-parametric Kruskal–Wallis test and Duncan’s multiple range test was used to identify differences between each group; the level of significance was set at *p* ≤ *0.05*^88^.

### Ethical approval and informed consent

Not applicable: This study does not directly involve humans or animals. Plant collection permits were not required because seed samples are commercial cultivars which can be purchased and no species are endangered or threatened.
